# Understanding the role of nurse practitioners, physician assistants and other nursing staff in HIV pre-exposure prophylaxis care in the United States: a systematic review and meta-analysis

**DOI:** 10.1186/s12912-020-00503-0

**Published:** 2020-12-09

**Authors:** Chen Zhang, Warton Mitchell, Ying Xue, Natalie LeBlanc, Yu Liu

**Affiliations:** 1 School of Nursing, University of Rochester Medical Center, 255 Crittenden Blvd., Rochester, New York 14622 USA; 2 School of Medicine and Dentistry, University of Rochester Medical Center, Rochester, New York USA

**Keywords:** Systematic review, Meta-analysis, PrEP care implementation, Non-physician health providers, United States

## Abstract

**Background:**

Although pre-exposure prophylaxis (PrEP) was approved for primary HIV prevention by the Federal Drug Administration in 2012, PrEP utilization has been suboptimal. A body of literature and programs has emerged to examine the role of nurse practitioners (NPs), physician assistants and nursing staff in PrEP care. This review aims to understand the current status of non-physician health providers in PrEP care implementation in the United States.

**Methods:**

Following the Preferred Reporting Items for Systematic Reviews and Meta-Analyses guidance, we conducted a comprehensive literature search using multiple databases to identify peer-reviewed articles that examined the role of non-physician health providers in the implementation of PrEP. Four major databases of studies using observational study design, randomized control trials and mixed-method study design were screened from November 2019 to January 2020 were searched. Two independent reviewers examined eligibility and conducted data extraction. We employed random-effects model aims to capture variances of estimates across studies.

**Results:**

A total of 26 studies with 15,789 health professionals, including NPs (18, 95% CI = 14,24%), physician assistants (6, 95% CI = 2, 10%), nursing staff (26, 95% CI = 18–34%), and physicians (62,95% CI = 45, 75%), were included in the analysis. The odds of prescribing PrEP to patients among NPs were 40% (OR = 1.40, 95% CI = 1.02,1.92) higher than that among physicians, while the likelihood of being willing to prescribe PrEP was similar. On the other hand, the odds of being aware of PrEP (OR = 0.63, 95% CI = 0.46, 0.87) was 37% less in nursing professionals than that among physicians.

**Conclusions:**

Although the limited number and scope of existing studies constrained the generalizability of our findings, the pattern of PrEP care implementation among non-physician health providers was described. To achieve wider PrEP care implementation in the U.S., increasing awareness of PrEP among all health providers including both physicians and non-physicians is a key step.

## Background

An estimated 1.1 million people in the United States (U.S.) are living with HIV [1]. The most heavily affected subgroups include men who have sex with men (MSM) of all races and ethnicities, with young black MSM shouldering a disproportionate burden, followed by Black and Latino heterosexuals [1, 2] and injection drug users [3]. HIV continues to burden these subgroups despite intensive behavioral and biomedical prevention efforts (e.g., HIV testing, condom promotion, syringe exchange programs) [4].

To curb the HIV epidemic and broaden HIV prevention strategies, the U.S. Food and Drug Administration approved the use of daily oral pre-exposure prophylaxis (PrEP; emtricitabine [FTC]/tenofovir disoproxil fumarate [TDF], brand name Truvada) in 2012. Persons at risk of HIV infection including MSM, persons at risk from heterosexual contact, and persons who inject drugs, and weighing at least 35 kg, should be considered for PrEP use (USPSTF) [5]. Later in 2019, another type of PrEP, Descovy, which contains a newer version of the medication, was approved by the FDA. Descovy has fewer side effects but with limited data for people at risk through receptive vaginal sex. Therefore, Descovy is recommended for HIV prevention among persons with the same indications as Truvada, but excluding people at risk through receptive vaginal sex (CDC) due to the lack of clinical trials [6]. Multiple randomized, placebo-controlled, clinical trials of PrEP have been conducted among high-risk MSM [7–9], transgender women [10], heterosexual men, and women [11, 12], serodiscordant heterosexual couples [13], and people who inject drugs [14]. The results concur that daily oral PrEP has desirable protective effectiveness (the relative risk of acquiring HIV was reduced by 39–75% among these at-risk groups) [4].

Although health professionals have made tremendous efforts, the uptake and provision of PrEP among people at risk of HIV acquisition has been slow, with only 9.0% of people with PrEP indications using it in 2018 [15]. Even with this small proportion of PrEP users, health inequity has been observed across different groups. For instance, there were 16 times more male PrEP users than female users in 2017, and PrEP users were predominately White [15]. To ensure health equity and curb the HIV epidemic, facilitating potential PrEP users with different characteristics to engage with PrEP care has become a top priority in both research and practice.

**Fig. 1:**
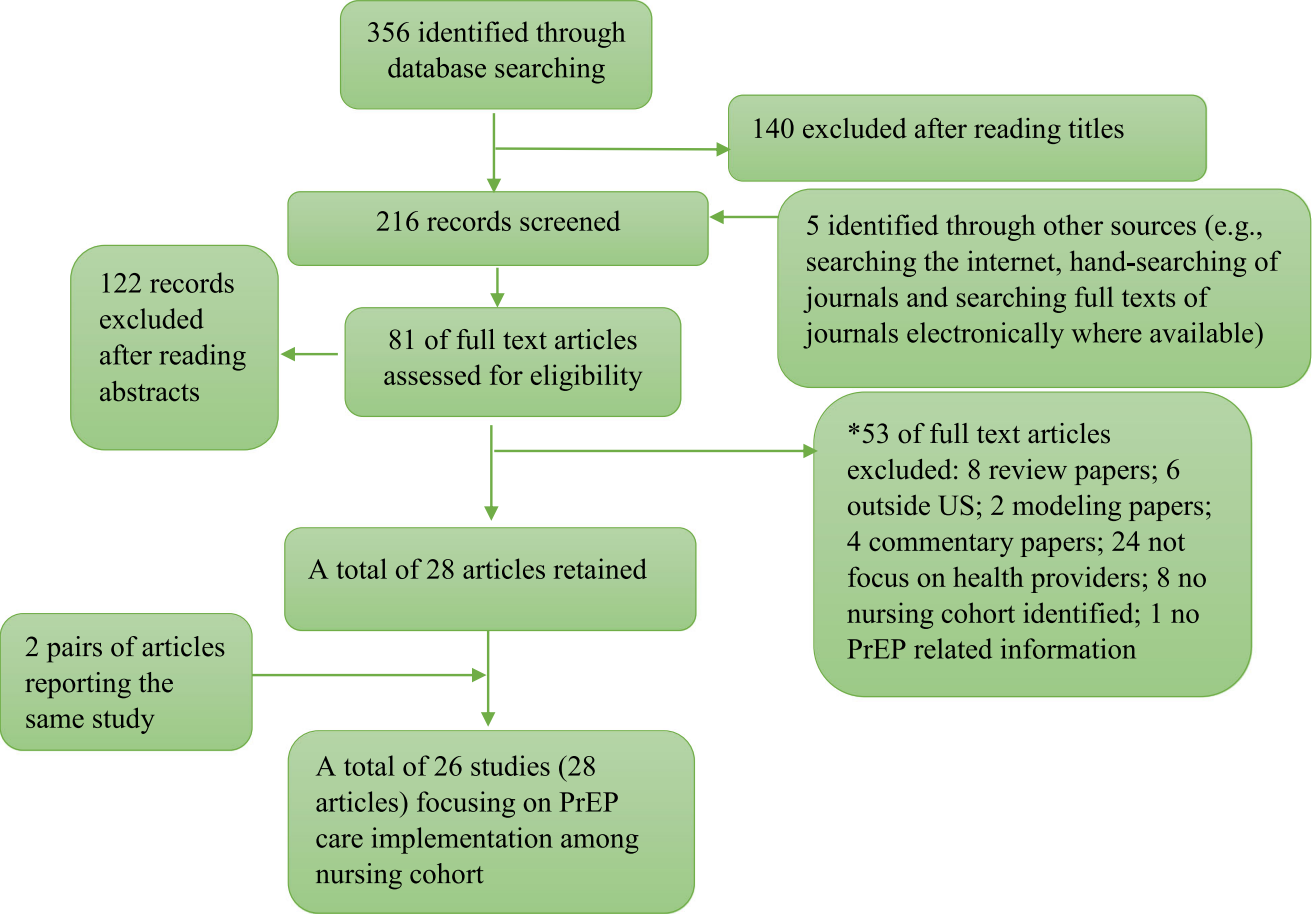
Flow Chart for study selection procedures. A total of 28 manuscripts (26 studies) were identified by January 28, 2020. Databases: PubMed/MEDLINE, Web of Science, PsycINFO, EMBASE, and Other sources (e.g., Google Scholar, Internet)

In this context, Nunn and colleagues proposed a PrEP care cascade model, which suggests that progression along stages of the cascade must involve interaction and engagement for both PrEP users and health providers (FigS1) [16]. A recent study described the role of health providers in terms of the PrEP care cascade and found 68% of them aware of PrEP, 66% willing to prescribe, and 24% ever prescribing [17]. However, the previous study did not distinguish from non-physicians from physicians, who may play different but essential roles in PrEP care. Among these non-physician health providers, nurse practitioners (NPs) constitute the most abundant and fast-growing group with prescriptive privileges in the healthcare workforce [18], especially in rural and low-income areas in the U.S. As the NPs widely employ the holistic biopsychosocial practice (i.e., exploring a patient’s biological, psychological, and social determinants of health outcomes), they have respectable reputations for providing effective and high-quality care, including PrEP care [19]. Furthermore, a growing body of literature that examined the role of NPs and nursing staff in PrEP care delivery has shown favorable outcomes [19]. In the current study, we assessed the specific role of non-physician health providers in the PrEP care cascade by synthesizing and analyzing available studies. We further quantitatively evaluated the reported barriers embedded within PrEP care.

## Methods

### Search strategy and selection criteria

The current review followed the guidance of the Preferred Reporting Items for Systematic Reviews and Meta-Analyses. Between November 2019 and January 2020, we conducted a comprehensive literature search from databases including PubMed/MEDLINE, Web of Science, PsycINFO, EMBASE, and other sources (e.g., google scholar, trial registers) with the following keywords: (1) HIV or AIDS; (2) PrEP care or PrEP implementation or PrEP care cascade; (3) health professionals or health providers; and (4) nursing cohort, nursing personnel, nursing professionals, advance nurse practitioners, or nurse practitioners (Table S1). A search for the reference list of included studies was also performed. Two reviewers (CZ and YL) independently reviewed the articles identified in the initial search, and disagreement was resolved by discussion. This review was registered on the PROSPERO website (CRD42020171527).

Published articles were included if they: (a) presented results on PrEP care implementation for a sample including NPs in the U.S.; (b) used rigorous study designs (e.g., randomized control trials, observational study designs, and mixed-method designs); (c) reported quantitative measures (e.g., proportions) for PrEP care cascade (e.g., PrEP awareness, willingness to prescribe PrEP, and PrEP prescription), or provided sufficient information to calculate pooled estimates or odds ratios; and (d) were peer-reviewed and published in English, and can be searched for in indexed databases or published sources. We excluded articles if they were: (a) studies without quantitative measurements; (b) studies that could not distinguish between nursing professionals and other types of health providers, (c) secondary research, and (d) modeling or commentary articles without original data. Where multiple articles reported a single cohort, only the article with the most comprehensive data was included.

### Data analysis

The pooled odds ratios of *PrEP awareness*, *willingness to prescribe PrEP*, and *PrEP prescription* between NPs and physicians were the key estimates in the current analyses. We also conducted subgroup analyses to examine these odds ratios in the PrEP cascade using study locations as the result of the uneven distribution of the current PrEP use across the nation. In addition, subgroup analyses were conducted based upon the time when the study was conducted due to the progress of PrEP use made across different years.

In addition to reporting crucial estimates, we reported distributions of included health providers by type (e.g., NPs, physicians) and specialty (i.e., infectious disease specialists vs. primary care providers). Their perceived barriers regarding the implementation of PrEP care (e.g., lack of knowledge, concern about toxicity, and side effects), as well as their perceived ideal locations for PrEP care (e.g., primary care settings, HIV clinics), were extracted and calculated.

A random-effects model aims to capture variances of estimates across studies [20, 21]. As the included studies were conducted among different populations at different settings, we would use random-effect model in meta-analysis with an assumption that the effects being estimated in the different studies are not identical. DerSimonian-Laird method has been widely validated in meta-analyses and shown fairly reliable approximation when the number of included studies is relatively large [22, 23]. In the analyses, the *metan* command was used to assess differences (odds ratios) in implementing each stage of PrEP care across provider types (e.g., nursing personnel vs. physicians)*.* Meanwhile, the *metaprop* command was used to pool the proportion data as it was mainly designed for data using the binomial distribution to model the within-study variances [24]. The pooled effect sizes incorporated each study’s weight, which was determined by the sample size of each individual study.

Additionally, sensitivity analyses were employed to examine the stability of the pooled estimates by evaluating whether the overall pooled estimates were sensitive to the exclusion of any individual studies (e.g., study with extreme weights or sample sizes). The *I*^*2*^*-statistics* describe the percentage of variability in effect estimates that is due to heterogeneity rather than sampling errors. As recommended by Borenstein et al. (2009) [25], the estimates of the degree of heterogeneity using overlapping intervals for I^2^-statistics were interpreted as unimportant (0–40%), moderate (30–60%), substantial (50–90%) and considerable (75–100%). The weights reflect the amount of information that each study contributes to the overall estimate, with a higher weight indicating a larger sample size [25]. Furthermore, we employed the GRADE rating scheme to evaluate the quality of evidence from each individual study using recommended criteria (i.e. risk of bias, precision, consistency, directness) [26]. We performed all statistical and meta-analysis using STATA®15 (College Station, TX).

### Role of funding source

The funder of the study had no role in study design, data collection, data analysis, data interpretation, or writing of the report. The corresponding author had full access to all the data in the study and had full responsibility in the final decision to submit the manuscript for publication.

## Results

The search using keywords yielded 356 results from the databases. After initial screening by reading titles, 216 were retained for further assessment. Another five articles were identified by 5 identified through other sources (e.g., searching the internet, hand-searching of journals, and searching full texts of journals electronically where available), resulting in a total of 216 records. These records were assessed by reading abstracts, which yielded 81 references reviewed for full-text. Of these, 53 articles were excluded for various reasons: eight were review papers, six were conducted outside the U.S., two were modeling papers, four were commentary papers, 24 were not focused on health providers, eight had no nursing professionals included, and one did not report PrEP care-related outcomes. A total of 28 articles met all inclusion criteria. Among all included publications, two pairs of articles reported the same data, and we retained only one from each pair with the most comprehensive information. In the end, a total of 26 studies reporting data regarding the PrEP care cascade were retained in the analysis (Fig. 1).

Detailed study characteristics (i.e., authors, location, time of the survey, recruitment, study design, and key measurements) are reported in Table S2. Although data collection spanned the period before and after 2012, all included studies were published in and after 2012, with sample sizes ranging from 15 to 9023 in included. The majority of studies employed cross-sectional study designs (*n* = 20). One study employed a longitudinal open cohort design to assess the PrEP implementation cascade across several years (e.g., 2009–2015) [27]. For studies using other types of study designs, five employed in-depth interviews and the rest used focus groups for data collection.

In the current analysis, a total of 15,789 U.S.-based health professionals, including physicians, NPs, physician assistants, registered nurses, and other types of health providers, were identified and included in the analysis. Specifically, the proportion of physicians was 62% (95% CI = 45, 75%), followed by 18% (95% CI = 14, 23%) of NPs, and 6% (95% CI = 2, 10%) of physician assistants. Among the included health providers, 44% (95% CI = 29, 60%) were primary care providers, and 30% (95% CI = 19, 42%) were infectious disease specialists (Table 1).

**Table 1:** Distribution of Advanced Nurse Practitioners, Physician Assistants, and all nursing staff by locations, study design and time of the study

	Types of Health Providers			Specialty of Health Providers	
	Nurse Practitioners	Physician assistants	All nursing staff	^a^PCP	^b^ID specialist
**Overall**	0.18 (0.13, 0.24)	0.06 (0.02, 0.10)	0.26 (0.18, 0.34)	0.44 (0.29, 0.60)	0.30 (0.19,0.42)
***I***^***2***^ **statistics**	98.47%	95.05%	99.15%	99.04%	98.64%
**By time of the study**					
< =year of 2012	0.13 (0.10, 0.17)	0.07 (0.03, 0.11)	0.30 (0.17, 0.45)	0.32 (0.25, 0.39)	0.28 (0.13, 0.47)
*Weights/ I*^*2*^*-statistics*	22.49%/ 84.87%	12.43%/ n/a^c^	29.75%/ 99.09%	12.22%/ n/a	27.50%/ 96.37%
> year of 2012	0.20 (0.13, 0.29)	0.06 (0.02, 0.11)	0.24 (0.15, 0.35)	0.45 (0.29, 0.62)	0.30 (0.17, 0.46)
*Weights/ I*^*2*^*-statistics*	77.51%/ 98.79%	87.57%/ 95.73%	70.25%/ 95.73%	87.78%/ 99.17%	72.50%/ 98.92%
Trend analysis	Prob > |z| = 0.956	Prob > |z| = 0.892	Prob > |z| =0.480	Prob > |z| = 0.407	Prob > |z| = 0.654
**By Location**					
Northeast	0.18 (0.08, 0.30)	0.06 (0.03, 0.10)	0.21 (0.14, 0.29)	0.52 (0.23, 0.81)	0.44 (0.23, 0.66)
*Weights/ I*^*2*^*-statistics*	25.29%/ 90.55%	43.03%/ 58.70%	22.49%/ 78.53%	30.39%/ 97.04%	27.08%/ 96.06%
South	0.14 (0.05, 0.33)	n/a^c^	0.60 (0.46, 0.73)	n/a^c^	0.14 (0.10, 0.19)
*Weights/ I*^*2*^*-statistics*	3.12%/ n/a^c^		13.09%/ 90.57%		16.77%/ n/a^c^
West	0.38 (0.35, 0.42)	0.00 (0.00, 0.01)	0.38 (0.35, 0.42)	0.41 (0.38, 0.45)	0.02 (0.01, 0.04)
*Weights/ I*^*2*^*-statistics*	7.98%/ n/a^c^	14.07%/ n/a^c^	6.80%/ n/a^c^	6.39%/ n/a^c^	11.24%/ n/a^c^
Midwest	0.94 (0.91, 0.96)	n/a^c^	1.00 (0.99, 1.00)	0.33 (0.28, 0.38)	n/a^c^
*Weights/ I*^*2*^*-statistics*	4.07%/ n/a^c^		3.44%/ n/a^c^	6.37%/ n/a^c^	
Nationwide	0.14 (0.11, 0.16)	0.07 (0.02, 0.15)	0.16 (0.12, 0.21)	0.42 (0.19, 0.66)	0.36 (0.23, 0.50)
*Weights/ I*^*2*^*-statistics*	59.54%/ 89.32%	42.90%/ 96.88%	54.19%/ 97.58%	56.85%/ 99.42%	44.91%/ 97.97%

^a^*PCP* Primary Care Providers, ^b^*ID* Infectious Disease, ^c^*n/a* Not Applicable

**Fig. 2:**
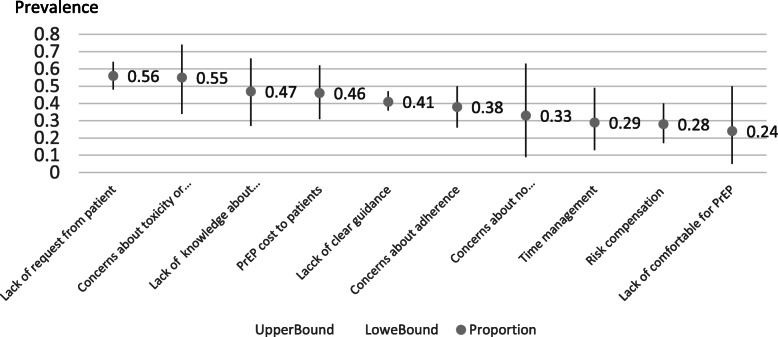
Health Providers’ Perceived Barriers duing PrEP Care Implementation

In the meta-analyses, we calculated the pooled odds ratios of each specific stage in the PrEP implementation cascade between NPs and physicians. The odds of being aware of PrEP among NPs were 37% (OR = 0.63, 95% CI = 0.46–0.87) less than that among physicians, while the odds of being willing to prescribe PrEP among NPs was not very different from that among physicians (OR = 1.00, 0.98, 1.02). On the other hand, the odds of prescribing PrEP were 1.40 (95% CI = 1.02. 1.92) times higher among NPs than that among physicians (FigS2a-c). We further assessed the odds ratios based on different times, locations, and study designs, and found that the odds ratio of prescription between NPs and physicians was highest in 2015 among all available studies (OR = 1.94, 95% CI = 1.12, 3.36) (Table 2).

**Table 2:** PrEP Awareness, Willingness to Prescribe, and Prescription Behaviors among ^@^Nurse Practitioners Compared with Physicians in the United States

	Odds Ratios and 95% CI	Heterogeneity chi-squared statistics	d.f.^a^	*I*^*2*^*-statistics*
	**PrEP Awareness**			
**Overall Awareness**	0.63 (0.46,0.87)	0.92	1	0.00%
***By Time***				
*2016*	0.63 (0.46,0.87)	n/a^b^	n/a^b^	n/a^b^
***By Location***				
*North East*	0.34 (0.1,1.156)	0.00	0	n/a^b^
*West*	0.63 (0.46,0.87)	0.00	0	n/a^b^
	**Willingness of prescribing PrEP**			
**Overall Willingness**	1.00 (0.98,1.02)	6.52	3	54.00%
***By Time***				
*2006*	0.74 (0.47,1.17)	n/a	n/a^b^	n/a^b^
*2014*	1.36 (0.65,2.86)	4.18*	1	76.10%
*2015*	1.00 (0.98,1.02)	n/a	n/a	n/a
***By Location***				
*North East*	0.78 (0.28,2.17)	n/a	n/a^b^	n/a^b^
*South*	0.74 (0.47,1.17)	n/a	n/a^b^	n/a^b^
*Nationwide*	1.00 (0.98,1.02)	3.24	1	69.10%
*West*	0.42 (0.11,1.62)	n/a	n/a^b^	n/a^b^
	**PrEP Prescription**			
**Overall Prescription**	1.40 (1.02,1.92)	8.99	4	55.50%
***By Time***				
*2013*	1.32 (0.89,1.97)	2.93	2	31.80%
*2014*	0.27 (0.06,1.21)	0.00	0	n/a^b^
*2015*	1.94 (1.12,3.36)	0.00	0	n/a^b^
***By Location***				
*North East*	2.59 (0.65,10.32)	0.00	0	n/a^b^
*Nationwide*	1.35 (0.98,1.87)	8.18*	3	63.30%

^**@**^Nurse practitioners: data were extracted directly from included studies where the category was labeled as “Nurse Practitioners”; *p < 0.05; ^a^*d* degree of freedom, ^b^*n/a* not applicable

**Fig. 3:**
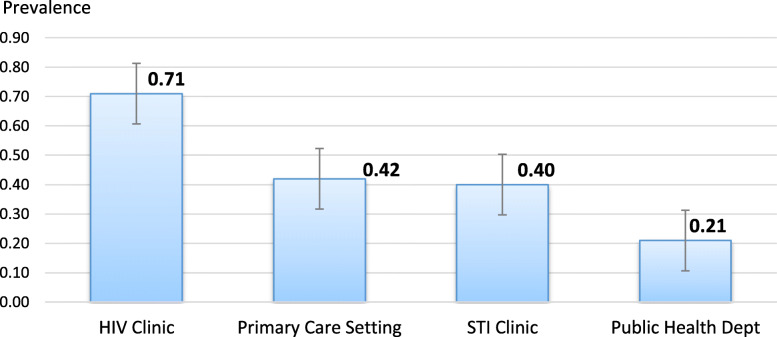
Reported Ideal Locations for PrEP Care among Health Care Providers

We further calculated pooled proportions of perceived barriers regarding PrEP care implementation among the included health providers. The most mentioned barrier regarding PrEP care was the lack of such a request from patients (56, 95% CI = 48–64%), followed by concerns about toxicity and resistance (55, 95% CI = 34, 74%), lack of knowledge about PrEP (47, 95% CI = 27, 66%), concern about the cost to patients (46, 95% CI = 31, 62%), lack of clear guidance (41, 95% CI = 36, 0.47%), concerns about patients’ adherence (38, 95% CI = 26, 50%), concerns about no supporting evidence (33, 95% CI = 9, 63%), time management (29, 95% CI = 13, 49%), risk compensation (28, 95% CI = 17, 40%), and lack of comfort regarding PrEP (24, 95% CI = 5, 50%) (Fig. 2). In addition, health providers reported that the ideal locations for PrEP care included HIV clinics (*p* = 71, 95% CI = 67, 74%), primary care settings (*p* = 42, 95% CI = 27, 58%), sexually transmitted disease clinics (*p* = 40, 95% CI = 8, 78%), and public health departments (*p* = 21, 95% CI = 16, 27%) (Fig. 3).

Heterogeneity, publication biases, risk of bias, and outlier assessment were all systematically assessed in the current study. Low to moderate between-study heterogeneity was detected across studies evaluating PrEP awareness (I^2^ = 0.00%), willingness (I^2^ = 54.00%), and prescription (I^2^ = 55.50%). Meanwhile, sensitivity analyses, including or excluding studies with extreme weights, showed no significant difference. The quality of evidence for most outcomes was scored as very low to moderate, primarily due to the nature of observational study design, limited sample size, and limited generalizability (Table S2).

## Discussion

Our study is the first to systematically review and synthesize data from the literature about the role of non-physician health providers that are composed of NPs, followed by other nursing staff and physician assistants, in PrEP care implementation in the U.S. Using GRADE, we assessed the certainty of the available evidence, and the overall quality of included studies were rated from very low to moderate, which are primarily due to the nature of cross-sectional study design or convenience sampling strategy.

We found that the odds of prescribing PrEP to patients with indications among NPs were higher than that among physicians. In contrast, the odds of being aware of PrEP among NPs are lower than their physician counterparts, which is consistent with the findings of a previous review study [17]. The top three most frequently reported barriers to PrEP care were consistent with findings from a recent synthesized analysis [17], including lack of request from patients, concerns about toxicity and resistance, and lack of knowledge about PrEP. Meanwhile, both HIV clinics and primary care settings are considered ideal locations for PrEP care. Our findings have crucial implications for expanding the role of non-physician health providers in PrEP care implementation by enhancing PrEP awareness and providing tailored and effective educational and clinical training. Our findings also elucidate directions for future research and intervention programs.

In the current study, we compared NPs and physicians in the specific stages of PrEP care implementation. Unlike most published studies that emphasize the role of physicians in PrEP care implementation [28–33], our study addressed both physicians and non-physician health providers including NPs. NPs have a long and successful history of delivering preventive and primary care to people in need [34], however, very few studies have assessed the specific role of NPs or other non-physician health providers in PrEP care in practice [19, 35]. A recent nationwide study showed that the supply of NPs increased more rapidly than physician supply, especially in marginalized or medically underserved areas, which can offset the shortage of physician supply in the U.S. [18] With the evolution of legislation to liberalize NPs’ scope of practice, NPs as well as other non-physician health providers, equipped with advanced tools (e.g., care coordination model, holistic approach) and clinical skills, can provide an efficient quality of care as their physician counterparts do [19, 36]. In the HIV Prevention Trials Network 073 study, advanced practice nurses, including NPs, were found to have successfully improved PrEP uptake and retention among 227 black MSM over 12 months [37]. Emerging evidence indicates that NPs are possibly in the best position for PrEP care practice [19].

Our study findings revealed that NPs were more likely to prescribe PrEP to patients with indications than physicians. Previous literature has examined prescribing practices between NPs and physicians on common medications. In general, prescribing patterns and quality were comparable between NPs and physicians [38]. Some studies found that NP prescription practices were different from physicians in rural areas and with regard to the prescription of opioid medication [39, 40]. Further studies are highly desired to assess the different practice patterns between non-physician health providers and physicians, especially among underserved populations or in underdeveloped areas.

Although the prevailing “purview paradox” debates (i.e., neither primary care providers nor infectious disease specialists consider that PrEP care should fall within their practice) have not been resolved yet, involving both primary care providers and infectious disease specialists may increase PrEP provision and improve PrEP care for people with different needs. Our data indicated that both primary care settings and HIV clinics were ideal locations for PrEP care. In addition, non-physician health providers with different expertise (i.e., primary care, infectious diseases, HIV) can play an essential role in both settings [19].

The lack of requests from patients ranked as the top reported barrier to PrEP care, and facilitating PrEP discussions may be a leeway for this barrier. Although studies have demonstrated that positive patient-provider relationships can foster patient engagement across HIV care and reduce health disparities across among individuals with different backgrounds [41], very few interventions are available to enhance communication skills and foster trust among health providers who are the gatekeepers of PrEP care [17, 42]. In addition, providers’ concerns regarding the toxicity and resistance of PrEP as well as the lack of PrEP knowledge revealed that insufficient support or training programs were available for healthcare providers regarding PrEP care.

Several strengths were identified in the current study. First, it is the first to assess the specific role of non-physician health providers in the PrEP care cascade as well as their perceived barriers and ideal locations of PrEP care. This study also contains a reasonably large number of studies (*n* = 26) with a considerable sample size (*n* = 15,789), affording it substantial power to detect outcomes [25]. Second, the DerSimonian-Laird random-effects modeling was employed to account for heterogeneity across studies [43]. We further used *metan* to calculate effect sizes and *meta prop* to calculate the pooled proportions based on the binominal nature of the data. Likewise, we filled a research gap by taking weighted sample sizes into consideration to control possibly inflated type I errors in published studies [44]. Meanwhile, a few caveats need to be taken into consideration when interpreting findings from the current study. First, only a part of the included studies examined the role of non-physician health providers in PrEP care in particular, and some studies categorized NPs and physician assistants or other nursing staff (e.g., registered or certified nurses) into one category, which may have constrained the precision and validity of key estimates among non-physician health providers due to the limited statistical power. Second because of the limited number of studies with moderate heterogeneity across studies, the pooled estimates along the PrEP cascade may be biased. Third, most included studies have relatively low quality due to the limited sample sizes and generalizability as well as the nature of the observational study designs, which indicate further trials are needed to rigorously assess the role of non-physician health providers in the PrEP care cascade.

## Conclusions

Utilizing synthesized data from 26 studies representing almost 16,000 health providers in the U.S., this is the first study to date to assess the role of non-physician health providers with pooled proportions in each stage of the PrEP implementation cascade. Considering that NPs are more likely to prescribe PrEP to patients with indications despite their lower PrEP awareness than physicians, our results shed light on accelerating the HIV PrEP scale-up through increasing PrEP awareness among these non-physician health providers. Non-physician health providers, including NPs, other nursing professionals and physician assistants, are essential assets to improve the PrEP care cascade and reduce health inequity by engaging vulnerable populations who are at risk of HIV infection, especially in disenfranchised areas in the U.S. Our findings can help guide future research and policy to address and close gaps in PrEP care. Moreover, as one of the key pillars in the strategic initiative proposed by the U.S. Department of Health and Human Services, PrEP plays a crucial role in ending the HIV epidemic in the United States [45]. Thus, only by understanding the role of non-physician health providers in the PrEP care cascade, can we design tailored interventions to engage them to optimize this critical strategy of interventions.

## Supplementary Information

**Additional file 1: Table S1.** Key searching terms for the current study (By January 282,020)1. **Table S2.** Key characteristics of included quantitative studies (*n* = 26). **FigS1.** PrEP Care Cascade Model. **FigS2a.** Forest plot of PrEP prescription of odds ratio between nurse practitioners and physicians. **FigS2b.** Forest plot for willingness of PrEP prescription of odds ratio between nurse practitioners and physicians. **FigS2c.** Forest plot PrEP awareness of odds ratio between nurse practitioners and physicians.

## Data Availability

The datasets used and/or analysed during the current study are available from the corresponding author on reasonable request.

## Abbreviations

MSM: Men who have sex with men
NPs: Nurse practitioners
PrEP: Pre-exposure prophylaxis
PRISMA: Preferred Reporting Items for Systematic Reviews and Meta-Analyses
