# Supplementary material for: Understanding the role of nurse practitioners, physician assistants and other nursing staff in HIV pre-exposure prophylaxis care in the United States: a systematic review and meta-analysis
Source: BMC Nurs. 2020 Dec 9;19:117. doi: 10.1186/s12912-020-00503-0 (PMC7724856; doi:10.1186/s12912-020-00503-0)
Supplement: Supplementary file 1 — Additional file 1: Table S1. Key searching terms for the current study (By January 282,020)1. Table S2. Key characteristics of included quantitative studies (n = 26). FigS1. PrEP Care Cascade Model. FigS2a. Forest plot of PrEP prescription of odds ratio between nurse practitioners and physicians. FigS2b. Forest plot for willingness of PrEP prescription of odds ratio between nurse practitioners and physicians. FigS2c. Forest plot PrEP awareness of odds ratio between nurse practitioners and physicians. [file 12912_2020_503_MOESM1_ESM.docx]

**Table S1. Key searching terms for the current study (By January 28 2020)^1,2^**

| **Search terms** | | **Search results** |
| --- | --- | --- |
| **#1** | "HIV"[Mesh] OR "HIV"[tiab] OR "HIV Infections"[Mesh] OR "HIV Antibodies"[Mesh] OR HIV-1[tiab] OR HIV1[tiab] OR HIV-2[tiab] OR HIV2[tiab] OR "HIV/AIDS"[tiab] OR "Sexually Transmitted Diseases, Viral"[Mesh:NoExp] OR "human immunodeficiency virus"[tiab] OR "human immunedeficiency virus"[tiab] OR "human immune deficiency virus"[tiab] OR "AIDS"[tiab] OR "acquired immunodeficiency syndrome"[tiab] OR "acquired immunedeficiency syndrome"[tiab] OR "acquired immune deficiency syndrome"[tiab] | [438327](https://www.ncbi.nlm.nih.gov/pubmed/?cmd=HistorySearch&querykey=1) |
| **#2** | "Pre-exposure prophylaxis"[Mesh] OR "PrEP"[MeSH] OR “Pre Exposure Prophylaxis”[tiab] OR “PrEP”[tiab] OR “Prophylaxi”[Mesh] OR “Prophylaxi”[tiab] OR “Pre-Exposure Prophylaxis (PrEP)”[tiab] OR “Pre Exposure Prophylaxis (PrEP)”[tiab] OR “Pre-Exposure Prophylaxi (PrEP)”[tiab] OR “Prophylaxi, Pre-Exposure (PrEP)”[tiab] OR “Prophylaxis, Pre-Exposure (PrEP)”[tiab] | [6006](https://www.ncbi.nlm.nih.gov/pubmed/?cmd=HistorySearch&querykey=2) |
| **#3** | “health care provider”[tiab] OR " health care workers "[Mesh] OR "providers"[tiab] OR "health professionals"[tiab] OR “health personnel” [tiab] OR "community health workers"[tiab] OR "family medicine providers"[ tiab] OR “obygn”[tiab] OR “OB-GYN”[tiab] OR “obstetrics & gynecology”[tiab] OR “reproductive providers”[tiab] OR “providers” [tiab] | [183267](https://www.ncbi.nlm.nih.gov/pubmed/?cmd=HistorySearch&querykey=3) |
| **#4** | "nurses"[tiab] OR “nurse practitioners”[tiab] OR " nursing cohort "[Mesh] OR "certified nurses"[tiab] OR "registered nurses"[tiab] OR “family nurse practiioners” [tiab] OR "community health workers"[tiab] OR "family medicine providers"[ tiab] OR "nurse midwives"[ tiab] OR “certified nurses”[tiab] OR “nurse clinicians”[tiab] | [186623](https://www.ncbi.nlm.nih.gov/pubmed/?cmd=HistorySearch&querykey=4) |
| **#5** | #1 AND #2 AND #3 | 339 |
| **#6** | #1 AND #2 AND #4 | 27 |

**Notes:** 1. Databases including PubMed/MEDLINE; 2. For Web of Science, PsycINFO, EMBASE, and others (e.g., Google Scholar), we used the key terms “HIV or AIDS”; “PrEP care or PrEP implementation or PrEP care cascade”; “health professionals or health providers”; and “nursing cohort, nursing personnel, nursing professionals, advance nurse practitioners, or nurse practitioners”

**Table S2. Key characteristics of included studies (n=26)**

| **Authors** | **Location** | **Time of survey** | **Characteristics of participants** | **No. of participants** | **Recruitment/Sampling regime** | **Study design** | **Key measurement** | ***GRADE rating** |
| --- | --- | --- | --- | --- | --- | --- | --- | --- |
| ^1^Adams 2016 | Mixed | June 2014 | HIV care providers with 47.3% being female | N=260 | Members of the American Academy of HIV Medicine (AAHVIM) were invited via e-mail to complete the survey/Convenience sampling | Cross-sectional | hypothetical willingness of prescription | ⊕⊕ΟΟ  Observational study design with convenience sampling, having limited representativeness; risk of bias is low or unclear |
| ^2^Bacon 2017 | San Francisco | May 2014 | A mixed group of HIV specialists and nonspecialists: aged 43 yrs (median); 56% female, 69% White | N=99 | A 20-item electronic, structured questionnaire was emailed to primary care providers in May 2014 through the 9-county San Francisco Bay Area Collaborative Research Network/Convenience sampling | Cross-sectional | PrEP knowledge, prior PrEP prescribing experience | ⊕⊕ΟΟ  Observational study design with limited representativeness; risk of bias is low or unclear |
| ^3^Bagchi 2018 | New Jersey | 2016 | Health care workers with 79.4% being female | N=174 | Online recruitment / convenience sampling | Cross-sectional | PrEP awareness and attitudes | ⊕⊕ΟΟ  Observational study design with limited sample size and representativeness; risk of bias is low |
| ^4^Blumenthal 2015 | New Yew and California | 2014 | HIV and non-HIV healthcare providers aged 40 (mean), 59% being White, and 60% being female | N=233 | Providers who work at the UCSD HIV or Infectious Diseases Clinics and regularly attend AIDS rounds but were not present when the study were offered were asked to complete the survey during their office hours. HIV providers in Los Angeles who are part of a Southern California research group with access to the iPads but with no prior knowledge of this study were also asked to complete the survey during their office hours /Convenience sampling | Cross-sectional | PrEP knowledge; PrEP perception; PrEP prescription experience | ⊕⊕ΟΟ  Observational study design with convenience sampling, having limited representativeness; risk of bias is low or unclear |
| ^5^Carter 2019/^6^Jayawardene 2018 | Indiana | 2017 | Advanced practice nurses with 93.8% being nurse practitioners and 95.4% being female | N=369 | Random sampling: An online questionnaire was fielded from March 2017 to May 2017 to a random sample of 1,358 APNs drawn from the total state population of 4,733 licensed APNs  with prescriptive authority; potential participants were identified through the Indiana licensure database | Cross-sectional | PrEP knowledge and beliefs | ⊕⊕ΟΟ  Observational study design with random sampling, having limited representativeness; risk of bias is low or unclear |
| ^7^Castel 2015 | Miami FL and Washington DC | March 2012-2013 | HIV providers in Miami FL and Washington DC, who had treated at least one HIV positive patients. | N=142 | Listing of HIV providers from physician societies, training centers, and health departments in both cities were used to identify potential participants/ Convenience sampling | Cross-sectional | PrEP knowledge, experience, and likelihood of prescribing | ⊕⊕ΟΟ  Observational study design with convenience sampling, having limited representativeness; risk of bias is low or unclear |
| ^8^Doblecki-lewis 2016 | South Florida | 2014 Oct.-Dec. | 6 medical providers, 7 administrators, and 9 case managers: 23% non-Hispanic White, all were at least with 2-yr of experience with HIV related patients | N=22 | Focus groups / convenience sampling | Focus group | Practical concerns and perceive limitations regarding PrEP implementation | ⊕⊕ΟΟ  Observational study design with convenience sampling, having limited representativeness; risk of bias is low or unclear |
| ^9^Finocchario-Kessler 2016 | Atlanta, Baltimore, Houston, Kansas City, Newark, Philadelphia, and San Francisco | 2013 Aug.-2014 Oct. | Health provider treating patients with HIV: 67.1% female, mean age of 43 yrs, 70.4% White. | N= 85 | Purposive sampling | Mixed methods (survey and in-depth interviews) | PrEP willingness and prescription experience | ⊕⊕⊕Ο  Observational study design with random sampling; risk of bias is low or unclear |
| ^10^Hakre 2016 | San Antoni, TX | 2015 | Primary care providers (PCP) and infectious disease physicians (ID) working at Air Force HIV Medical Evaluation Unite, with mean age of 36.7yrs, 41% being female and 74% being White | N=403 | The director of the Air Force HIV Medical Evaluation Unit contacted active duty primary care providers and infectious disease physicians by email with an invitation to participate in a web-based needs assessment survey/ Convenience sampling | Cross-sectional | Experience and willingness of PrEP provision | ⊕⊕ΟΟ  Observational study design with convenience sampling, having limited representativeness; risk of bias is low or unclear |
| ^11^Hart-Cooper 2018 | Nationwide | 2016 | Clinicians who work with adolescents and young adults | N=162 | An online survey was sent via the Society of Adolescent Health and Medicine | Cross-sectional | PrEP willingness | ⊕⊕ΟΟ  Observational study design with convenience sampling, having limited representativeness; risk of bias is low |
| ^12^Hoffman 2016 | New York City regions | Late 2012-early 2013 | Of the 30 participants, 24 were physicians, four were nurse practitioners, and two were physician assistants | N=30 | purposive sampling | In-depth interview | PrEP prescription experience, anticipated challenges, intention to implement PrEP care, training needs | ⊕ΟΟΟ  Observational study design with a very small sample size; risk of bias is low or unclear |
| ^13^Krakower 2016 | Boston, MA | 2015 | Primary care clinicians with mean age of 37 yrs, 53% being female and 81% being White | N=32 | All PCPs (n=35) at Fenway Health were invited to complete anonymous 35-item surveys assessing experiences with PrEP provision/Convenience sampling | Cross-sectional | Experience and willingness of PrEP provision | ⊕ΟΟΟ  Observational study design with a very small sample size; risk of bias is low or unclear |
| ^14^Krakower 2017 | Boston/MA | 2013 Sep-2014 Aug | 12 PCPs from LGBT specialist and 19 PCPS from a general academic medical center | N=31 | purposive sampling | In-depth interview | how they approach decisions about prescribing PrEP to MSM and their experiences with PrEP provision | ⊕ΟΟΟ  Observational study design with a very small sample size; risk of bias is low or unclear |
| ^15^Krawkower 2015 | New England | September-December 2013 | Health care providers in New England: age (mean=44yrs), 58% were female and 82% were White. | N=184 | Healthcare practitioners affiliated with a regional AIDS Education and Training Center in New England were invited to complete online surveys/Convenience sampling | Cross-sectional | PrEP awareness and prescription, willingness of PrEP prescription | ⊕⊕ΟΟ  Observational study design with convenience sampling, having limited representativeness; risk of bias is low |
| ^16,17^Mullins 2015/2016 | Nationwide | 2012 -2013 | US clinicians caring for HIV-infected and at-risk youth. | N=15 | convince sampling | In-depth interview | Belief about the role of PrEP in HIV prevention for adolescents, perceived barriers/facilitators regarding PrEP care | ⊕ΟΟΟ  Observational study design with a very small sample size; risk of bias is low |
| ^18^Mullins 2017 | 14 US locations | January  and April 2014 | Clinicians who were based at one of the14 US locations and provided care to HIV-infected youth: age= 45.9 yrs (sd= 10.7); half (n = 27. 5%) self-described as non-Hispanic White; 43 (77%) reported female gender at birth(n = 36; 64%) were physicians | N=56 | Online survey: participants  were recruited through the National Institutes of Health funded Adolescent Medicine Trials Network for HIV/AIDS  Interventions (ATN) /Convenience sampling | Cross-sectional | Intention to prescribe PrEP; actual prescription of PrEP to adults and adolescents | ⊕ΟΟΟ  Observational study design with a very small sample size; risk of bias is low or unclear |
| ^19^Petroll 2017 | 10 US locations | July 2014 to May 2015 | Primary care providers and HIV providers | N=525 | Online survey: potential participants were recruited via national databases including American Medical Association, American Association of Nurse Practitioners, and American Academy of HIV Medicine/ Convenience sampling | Cross-sectional | PrEP attitudes and barriers for PrEP care | ⊕⊕ΟΟ  Observational study design with convenience sampling, having limited representativeness; risk of bias is low |
| ^20^Przybyla 2019 |  | September 2017 to January 2018 | Current PrEP prescribers | N=28 | Convenience sampling | In-depth interview | Clinicians’ perspectives on PrEP practice | ⊕ΟΟΟ  Observational study design with a very small sample size; risk of bias is low or unclear |
| ^21^Scherer 2014 | New York City | 2013 October | New York area care providers for PLWHA: 71% (n=71) are female; 60% are white (n=87); age (mean)=50 (24-69) | N=145 | Distributed during a HIV related conference/ sampling | Cross sectional | Attitudes, knowledge and practice pattern toward fertility and conception in serodiscordant couples | ⊕⊕ΟΟ  Observational study design with convenience sampling, having limited representativeness; risk of bias is low or unclear |
| ^22^Seidman 2016 | Mixed | 2015 | Family planning providers: being female (95%), being white (81%),being a nurse practitioner or physician assistant (53%), being 55 years old and over (42%) and seeing 20 or more patients per week (58%) | N=495 | Family planning providers recruited via website postings, national meetings, and email completed an anonymous survey/Convenience sampling | Cross-sectional | PrEP prescribing experience | ⊕⊕ΟΟ  Observational study design with convenience sampling, having limited representativeness; risk of bias is low or unclear |
| ^23^Smith 2015 | Mixed | 2009-2015 | Physicians and nurse practitioners were surveyed in 2009-2015 | N=1500(2009); N=1504(2010); N=1503(2012); N=251(2012, pharmacist); N=1507(2013); N=1508(2014); N=1751(2015) | Web-based surveys with a main sample of primary care physicians and additional samples of other selected specialties/Convenience sampling | Cross-sectional | Willingness and prescription of PrEP | ⊕⊕⊕Ο  Observational study design with random sampling; risk of bias is low |
| ^24^Tellalian 2013 | Mixed | April-September 2011 | Members of American Academy of HIV Medicine (AAHIVM), with 44% females | N=189 | Survey was sent to members of AAHIVM/convenience sampling | Cross-sectional | Knowledge, perceptions, attitudes, prescription, and concerns about PrEP | ⊕⊕ΟΟ  Observational study design with convenience sampling, having limited representativeness; risk of bias is low or unclear |
| ^25^Tripathi 2012 | South Carolina and Mississippi | September 2006  -January 2008 | Providers from sexually transmitted disease and family planning clinics: Median age was 46.9 years and a majority were women (279 [78%]), non-Hispanic white (277 [78%]), non-physicians (254 [71%]), and public health care providers (223 [62%]). | N=360 | The final survey was mailed to 480 providers at sexually transmitted disease and family planning care settings/Convenience sampling | Cross sectional | PrEP knowledge; hypothetical willingness of prescribing PrEP | ⊕⊕ΟΟ  Observational study design with convenience sampling, having limited representativeness; risk of bias is low or unclear |
| ^19,26^Walsh 2017/Petroll 2017 | Mixed | July 2014-May 2015 | A total of 280 PCPs were included in the analytical analyses with a mean age of 50 (SD=8), 56% White and 48% of women. | N=280 | Potential participants were recruited using databases from three professional organizations. Only providers who practices in the ten U.S. cities with largest number of HIV cases were recruited. Providers should be within zip code where HIV prevalence was at least 0.5%./Purposive sampling | Cross-sectional | PrEP awareness, prescription and discussion with patients regarding PrEP | ⊕⊕ΟΟ  Observational study design with convenience sampling, having limited representativeness; risk of bias is low or unclear |
| ^27^Weiser 2017 | Mixed | 2013-2014 | Nationwide HIV care providers, with 41% being female, and 64% being White | N=935 | Data describing provider characteristics and practices were obtained from the 2013–2014 Medical Monitoring Project Provider Survey/ Probability sampling | Cross-sectional | PrEP prescribing experience | ⊕⊕⊕Ο  Observational study design with random sampling; risk of bias is low |
| ^28^Wood 2018 | Mixed | May 2016 | Licensed medical providers from WA state Department of Health | N=735 | Survey was sent based upon email list of licensed health providers at WA/ convenience sampling | Cross-sectional | PrEP knowledge, attitudes, prescription experience and identify implementation barriers | ⊕⊕ΟΟ  Observational study design with convenience sampling, having limited representativeness; risk of bias is low or unclear |

**Notes: ***GRADE rating for quality of evidence: ⊕ΟΟΟ-very low quality; ⊕⊕ΟΟ-low quality; ⊕⊕⊕Ο-moderate quality

**References**

1. Adams LM, Balderson BH. HIV providers' likelihood to prescribe pre-exposure prophylaxis (PrEP) for HIV prevention differs by patient type: a short report. *AIDS care.* Sep 2016;28(9):1154-1158.

2. Bacon O, Gonzalez R, Andrew E, et al. Brief Report: Informing Strategies to Build PrEP Capacity Among San Francisco Bay Area Clinicians. *Journal of acquired immune deficiency syndromes (1999).* Feb 1 2017;74(2):175-179.

3. Bagchi AD, Holzemer W. Support for PrEP Among New Jersey Health Care Workers. *The Journal of the Association of Nurses in AIDS Care : JANAC.* Nov - Dec 2018;29(6):849-857.

4. Blumenthal J, Jain S, Krakower D, et al. Knowledge is Power! Increased Provider Knowledge Scores Regarding Pre-exposure Prophylaxis (PrEP) are Associated with Higher Rates of PrEP Prescription and Future Intent to Prescribe PrEP. *AIDS and behavior.* May 2015;19(5):802-810.

5. Carter GA, Jayawardene W, Agley J, et al. Development of a 10-Item Tool to Identify Advanced Practice Nurse Readiness to Prescribe Pre-exposure Prophylaxis. *The Journal of the Association of Nurses in AIDS Care : JANAC.* May-Jun 2019;30(3):312-320.

6. Jayawardene W, Carter G, Agley J, Meyerson B, Garcia JR, Miller W. HIV pre-exposure prophylaxis uptake by advanced practice nurses: Interplay of agency, community and attitudinal factors. *Journal of advanced nursing.* Nov 2019;75(11):2559-2569.

7. Castel AD, Feaster DJ, Tang W, et al. Understanding HIV Care Provider Attitudes Regarding Intentions to Prescribe PrEP. *Journal of acquired immune deficiency syndromes (1999).* Dec 15 2015;70(5):520-528.

8. Doblecki-Lewis S, Liu A, Feaster D, et al. Healthcare Access and PrEP Continuation in San Francisco and Miami After the US PrEP Demo Project. *Journal of acquired immune deficiency syndromes (1999).* Apr 15 2017;74(5):531-538.

9. Finocchario-Kessler S, Champassak S, Hoyt MJ, et al. Pre-Exposure Prophylaxis (PrEP) for Safer Conception Among Serodifferent Couples: Findings from Healthcare Providers Serving Patients with HIV in Seven US Cities. *AIDS patient care and STDs.* Mar 2016;30(3):125-133.

10. Hakre S, Blaylock JM, Dawson P, et al. Knowledge, attitudes, and beliefs about HIV pre-exposure prophylaxis among US Air Force Health Care Providers. *Medicine.* Aug 2016;95(32):e4511.

11. Hart-Cooper GD, Allen I, Irwin CE, Jr., Scott H. Adolescent Health Providers' Willingness to Prescribe Pre-Exposure Prophylaxis (PrEP) to Youth at Risk of HIV Infection in the United States. *The Journal of adolescent health : official publication of the Society for Adolescent Medicine.* Aug 2018;63(2):242-244.

12. Hoffman S, Guidry JA, Collier KL, et al. A Clinical Home for Preexposure Prophylaxis: Diverse Health Care Providers' Perspectives on the "Purview Paradox". *Journal of the International Association of Providers of AIDS Care.* Jan-Feb 2016;15(1):59-65.

13. Krakower DS, Maloney KM, Grasso C, Melbourne K, Mayer KH. Primary care clinicians' experiences prescribing HIV pre-exposure prophylaxis at a specialized community health centre in Boston: lessons from early adopters. *Journal of the International AIDS Society.* 2016;19(1):21165.

14. Krakower DS, Ware NC, Maloney KM, Wilson IB, Wong JB, Mayer KH. Differing Experiences with Pre-Exposure Prophylaxis in Boston Among Lesbian, Gay, Bisexual, and Transgender Specialists and Generalists in Primary Care: Implications for Scale-Up. *AIDS patient care and STDs.* Jul 2017;31(7):297-304.

15. Krakower DS, Oldenburg CE, Mitty JA, et al. Knowledge, Beliefs and Practices Regarding Antiretroviral Medications for HIV Prevention: Results from a Survey of Healthcare Providers in New England. *PloS one.* 2015;10(7):e0132398.

16. Mullins TL, Lally M, Zimet G, Kahn JA. Clinician attitudes toward CDC interim pre-exposure prophylaxis (PrEP) guidance and operationalizing PrEP for adolescents. *AIDS patient care and STDs.* Apr 2015;29(4):193-203.

17. Mullins TL, Zimet G, Lally M, Kahn JA. Adolescent Human Immunodeficiency Virus Care Providers' Attitudes Toward the Use of Oral Pre-Exposure Prophylaxis in Youth. *AIDS patient care and STDs.* Jul 2016;30(7):339-348.

18. Mullins TLK, Zimet G, Lally M, Xu J, Thornton S, Kahn JA. HIV Care Providers' Intentions to Prescribe and Actual Prescription of Pre-Exposure Prophylaxis to At-Risk Adolescents and Adults. *AIDS patient care and STDs.* Dec 2017;31(12):504-516.

19. Petroll AE, Walsh JL, Owczarzak JL, McAuliffe TL, Bogart LM, Kelly JA. PrEP Awareness, Familiarity, Comfort, and Prescribing Experience among US Primary Care Providers and HIV Specialists. *AIDS and behavior.* May 2017;21(5):1256-1267.

20. Przybyla S, LaValley S, St Vil N. Health Care Provider Perspectives on Pre-exposure Prophylaxis: A Qualitative Study. *The Journal of the Association of Nurses in AIDS Care : JANAC.* Apr 3 2019.

21. Scherer ML, Douglas NC, Churnet BH, et al. Survey of HIV care providers on management of HIV serodiscordant couples - assessment of attitudes, knowledge, and practices. *AIDS care.* 2014;26(11):1435-1439.

22. Seidman D, Carlson K, Weber S, Witt J, Kelly PJ. United States family planning providers' knowledge of and attitudes towards preexposure prophylaxis for HIV prevention: a national survey. *Contraception.* May 2016;93(5):463-469.

23. Smith DK, Van Handel M, Wolitski RJ, et al. Vital Signs: Estimated Percentages and Numbers of Adults with Indications for Preexposure Prophylaxis to Prevent HIV Acquisition--United States, 2015. *Journal of the Mississippi State Medical Association.* Dec 2015;56(12):364-371.

24. Tellalian D, Maznavi K, Bredeek UF, Hardy WD. Pre-exposure prophylaxis (PrEP) for HIV infection: results of a survey of HIV healthcare providers evaluating their knowledge, attitudes, and prescribing practices. *AIDS patient care and STDs.* Oct 2013;27(10):553-559.

25. Tripathi A, Ogbuanu C, Monger M, Gibson JJ, Duffus WA. Preexposure prophylaxis for HIV infection: healthcare providers' knowledge, perception, and willingness to adopt future implementation in the southern US. *Southern medical journal.* Apr 2012;105(4):199-206.

26. Walsh JL, Petroll AE. Factors Related to Pre-exposure Prophylaxis Prescription by U.S. Primary Care Physicians. *American journal of preventive medicine.* Jun 2017;52(6):e165-e172.

27. Weiser J, Garg S, Beer L, Skarbinski J. Prescribing of Human Immunodeficiency Virus (HIV) Pre-exposure Prophylaxis by HIV Medical Providers in the United States, 2013-2014. *Open forum infectious diseases.* Winter 2017;4(1):ofx003.

28. Wood BR, McMahan VM, Naismith K, Stockton JB, Delaney LA, Stekler JD. Knowledge, Practices, and Barriers to HIV Preexposure Prophylaxis Prescribing Among Washington State Medical Providers. *Sexually transmitted diseases.* Jul 2018;45(7):452-458.

# **FigS1. PrEP Care Cascade Model (Cited from Nunn et al., (2017). Defining the HIV pre-exposure prophylaxis care continuum)**

**Notes:** The PrEP Care Continuum figure illustrates tht progression along stages of the cascade. With several key stages (identify individuals at highest HIV risk, link to PrEP care, prescribe PrEP, PrEP uptake, retention and adherence), it must involve interaction and engagement for both PrEP users and health providers.

**FigS2a. Forest plot of PrEP prescription of odds ratio between nurse practitioners and physicians**

**FigS2b. Forest plot for willingness of PrEP prescription of odds ratio between nurse practitioners and physicians**

**FigS2c. Forest plot PrEP awareness of odds ratio between nurse practitioners and physicians**
